# I’ve *Gut* A Feeling: Microbiota Impacting the Conceptual and Experimental Perspectives of Personalized Medicine

**DOI:** 10.3390/ijms19123756

**Published:** 2018-11-27

**Authors:** Amedeo Amedei, Federico Boem

**Affiliations:** 1 Department of Experimental and Clinical Medicine, University of Florence, Largo Brambilla, 03 50134, Firenze, Italy; federico.boem@gmail.com; 2 Department of Biomedicine, Azienda Ospedaliera Universitaria Careggi (AOUC), Largo Brambilla, 03 50134, Firenze, Italy

**Keywords:** microbiome, health, precision medicine, genomics

## Abstract

In recent years, the human microbiota has gained increasing relevance both in research and clinical fields. Increasing studies seem to suggest the centrality of the microbiota and its composition both in the development and maintenance of what we call “health” and in generating and/or favoring (those cases in which the microbiota’s complex relational architecture is dysregulated) the onset of pathological conditions. The complex relationships between the microbiota and human beings, which invest core notions of biomedicine such as “health” and “individual,” do concern not only problems of an empirical nature but seem to require the need to adopt new concepts and new perspectives in order to be properly analysed and utilized, especially for their therapeutic implementation. In this contribution we report and discuss some of the theoretical proposals and innovations (from the ecological component to the notion of polygenomic organism) aimed at producing this change of perspective. In conclusion, we summarily analyze what impact and what new challenges these new approaches might have on personalized/person centred/precision medicine.

## 1. Introduction

A famous metaphor to describe “life” is the tree. The tree enables to express two life aspects, that might appear somehow in contrast. On the one hand, each leaf stands for a species, highlighting a particular unique form according to which “life” manifests itself, thus showing the differences among living things and justifying the need for classification. On the other hand, each branch, connecting species, represents the historical trajectory (roughly saying: the phylogeny) recalling the fact that all living creatures share a common descent and reminding us that our partition of the living world is not so cutting as we would like. The tension between the necessity to order the living world and the awareness of its “ramified” unity, is a key feature of biological sciences since their origin [1]. Biological species are surely grasped and described as distinct one from each other, however not sharply as the categories that we usually adopt to classify them. Species boundaries are, in many cases, fuzzy rather than sharp. Indeed, biological species do not exist (in the real world) in the isolation of taxonomical hierarchies. They are rather intimately connected to each other. Sometimes the nature of this relation is so intrinsic that is labelled as symbiosis or the reciprocal interdependence of different organisms (either parasitic or mutualistic). So, human beings and their microbial community can be seen as a clear example of symbiosis.

However, the fuzziness of biological borders can be considered also from a different angle. New findings, also made possible by a new theoretical perspective, challenge the notion of *symbiosis* as such and push towards a radical change in the organization of the living world. Life is less linear and far more intricate than we thought. Recent evidence indicate that *horizontal gene transfer*, which is a form of transfer of genetic material that does not occur vertically (from parents to offspring) but horizontally and therefore it is also called *lateral gene transfer* [2], can be widespread and a broader phenomenon than thought (not confined to prokaryotes and thus including eukaryotes). Phylogeny is no longer only a vertical trajectory and of note, evolution can operate also horizontally. Maybe, a more suitable metaphor than the *tree,* describing this aspect of life is constituted by the *web*. According to some researchers, individuals belonging to plant or animal species should no longer be considered as such, that is, as single, distinctive biological forms, but rather as networks of biomolecular interactions, whose nodes are represented by the host and its associated microorganisms. These networks, to all effects new categories of biological organization, are called *holobionts*. Given the nature of these relationships, the holobionts’ genomes should be treated together and not separately, thus constituting a *hologenome*. Such intergenomic associations become so essential that previous models of animal and plant biology, without this dimension, should be considered, at least, partial and sketchy [3].

The consequences of such a change, that is conceptual before being experimental, have the potential to radically transform not just the way we use to understand various biomedical phenomena (such as certain physiological functions or dysfunctions) but also the modalities through which we could interfere with the very same phenomena (*e.g.,* which therapies).

Given such a new perspective, it should not be surprising that human *microbiota* (usually meant the variety of “microbial taxa associated with humans” [4]) has become the pivot of an intense investigation. The nature and the modalities of these association might vary, depending on the functions and mechanisms considered, but it is now widely recognized that the relationship between microbial communities and their host is fundamental both for basic and applied research (especially biomedical) [5].

**Figure 1:**
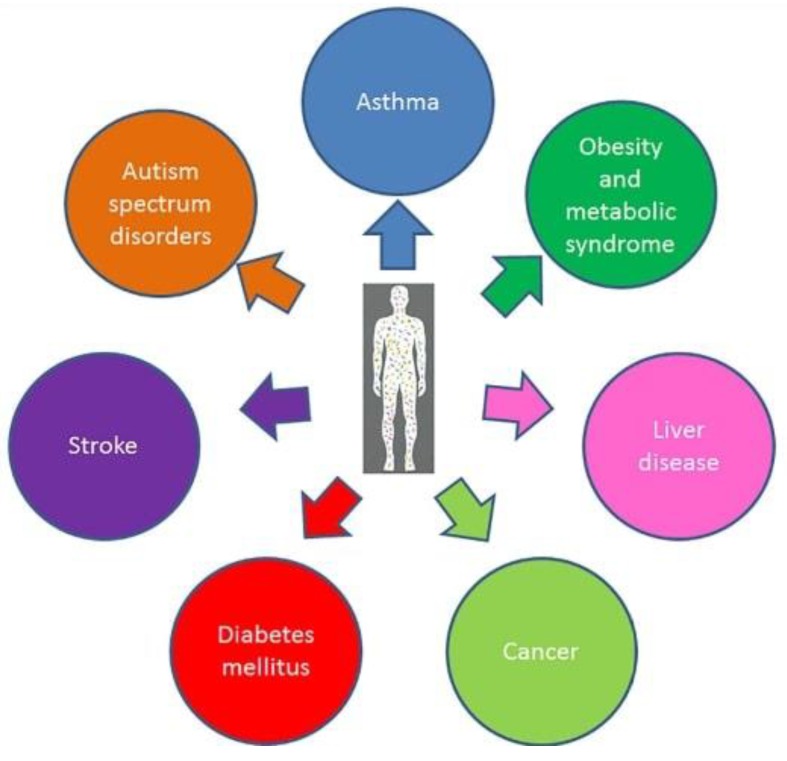
Different Human diseases correlated with the gut microbiome. We have reported some of the medical conditions where the experimental data suggest a direct involvement of gut microbiome in development.

New evidence highlight how microbiota plays a key role in human physiology, directly affecting metabolic pathways, spanning from intestinal to brain activities [6,7]. Indeed, recent findings indicate how an imbalance in the architecture of intestinal microbial populations might be directly involved in the development of different medical conditions (from metabolic to mood disorders) shedding new light on the aetiology of different diseases (such as obesity, asthma, autism spectrum disorders, stroke, diabetes and cancer) [6] (Figure 1).

Increasing studies suggest that microbiota contributes, in different ways and through different modalities, to the thin “red line” that separates physiological conditions from pathological ones. Recently, some researchers have acknowledged the central role of different bacterial populations and strains in modulating the adaptive immune response, thus affecting, for example, cancer development [8]. Moreover, a more precise understanding of general microbiota composition, imbalance among and within bacterial populations and their different localizations (*e.g.,* either gut residents or oral ones) may offer new hints to understand the origin and the progression of certain disease and thus new potential tools for a more specific diagnosis [9].

This situation is also due to the magnitude (both functionally and structurally) of microbiota itself, given that microbial cells in the gut outnumber cells of the host [6]. Microbiota metabolites and especially SCFAs (Short-Chain Fatty Acids) connect different areas of the organism, through the mediation of the immune and hormone system, such as the so called *gut-brain axis* [6,10,11] suggesting that the crosstalk between the organism and its microbial residents is a crucial factor for the sustenance of physiological and health conditions. In addition to that, it is imperative to recall that these microorganisms are no longer localized just in the gut. This can explain why the transition, within scientific terminology, from “gut flora” to “microbiota” does not coincide just with the need of a more precise or less restrictive, semantics. Rather, as words count for concepts, such a shift mirrors the fact that commensal, non-commensal and pathogenic organisms populate (beyond the intestinal tract) the skin, oral mucosa, lungs and other organs and tissues of the so-called “host organism” [12]. In addition, resident microorganisms are not just bacteria: fungi, phages and even viruses definitely belong to the microbiota broadly intended and constitute some of its new genuine subdivisions.

Finally an important caveat. The growing number of studies on the microbiota has generated many hopes, expectations but also a sort of *explanatory hype*, that would make the microbiota the new keystone for the understanding of otherwise unexplainable phenomena [13]. This vision is not only simplistic and reductive but also dangerous. The difficulty in establishing causal directions and priorities in biology suggests caution and urges us to consider the growing impact of studies on the microbiota in another light. Microbiota is indeed central but is a pivotal element among others. Therefore, in describing and reporting the theoretical proposals concerning the study and understanding of the microbiota, it is necessary to remember how the increasing interest on it should not be taken as privileged one compared to others. Rather, new perspectives on it should be seen as a way to build new systemic approaches (in which, for example, human and microbial genetics are considered more closely related). Hopefully, these systemic approaches would provide a more refined and adequate basis for personalized medicine

## 2. Composition and Microbiota Status

Due to computational methods, such as metagenomic sequencing [14], it is now possible to assess the genetic contribution of bacteria to host’s activities and to obtain a better (although still not exhaustive and incomplete) estimation of microbial population. To the most recent status of knowledge, microbial community is constituted by a biomass of 1.5–2.0 kg, mainly composed by anaerobic *Bacteria* [6]. According to recent reviews [6,15] the most represented phyla are *Actinobacteria*, *Bacteroidetes*, *Firmicutes*, *Proteobacteria* and *Verrucomicrobia*. The composition diversity and the balance between different phyla and populations (among and within phyla) may differ from the different subjects. Nevertheless, these differences, maybe in distinct ways and by distinct means, see some key functions preserved (such as degradation of some chemicals) in order to display and maintain “normal and physiological conditions.” However, it is always very hard to determine what is “normal” in a biological sense. A thing that might be unfeasible for an individual, could be on average for another one. As a matter of fact, the complex relationship existing between microbial interactions and composition and other factors (such as, among the others, the human immune system) is a key factor to determine not just a more adequate notion of *health* in general but, also, a more adequate notion of “*personal health”* (*i.e*., its unique conditions). Moreover, as already mentioned, microbiota cannot be reduced or restricted to anaerobic Bacteria. The very same development of sequencing technologies revealed that other organisms, less investigated, might have a (more or less impactful) role in this complex network of interactions. For instance, the term “Mycobiota*”* [16] has been then coined to address the fungal component (most of the time composed by not culturable strains) of the general microbiota. Similar studies have started to be conducted concerning *viral* genomes [17], sometimes defined as “Virome*”*).

In addition, one must not forget that biological landscapes as such are shaped by the interaction with the environment. This does not refer just to general, external conditions (*e.g.,* the pH) but of course, involve both dietary and life style habits of the “host” and its genome/epigenome. Both commensal and non-commensal organisms (among the same or different population and phyla) can either collaborate or compete to niche construction/shape creating a true, intricate *ecosystem*. If we embrace the perspective of the human body as an “ecosystem,” then ecological/relational categories and new theoretical tools [18,19] will be needed to correctly address this picture (potentially modifying or updating the notion of health itself).

As a consequence of the microbiota importance in all the aforementioned aspects of humans biology, several scholars and scientists have elaborated new ways and proposals to adequately address this archetype. In this review we want to examine these different proposals (from the *missing organ hypothesis* to the *polygenomic organism*), which have been developed with the purpose of elaborating conceptual tools that could account for the current developments of scientific investigations. In fact, some of the last recent research directions have highlighted that central concepts of contemporary biology such as organism, organ, symbiosis and so forth, seem to be no longer perfectly adequate, given the time of their formulation, to account for the latest findings and discoveries produced by experimental work. This set of theoretical elaborations, which we will briefly present here, appears central and actually responds to a twofold movement. On the one hand, as mentioned, new concepts are necessary in order to be able to describe and substantiate, more accurately, the new types of phenomena that research discovers. On the other hand, a new conceptual and theoretical equipment is precisely what makes the discovery and classification of new phenomena possible.

## 3. Microbiota: the Missing Organ

Due to its relevance for human health and physiology, some researchers have proposed to refer to microbiota as a “missing organ” of human body [20,21,22,23]. Far from being just an analogy or a working metaphor, these authors declare that there are strong reasons to look at microbiota as a true organ. From a functionalist perspective, it is argued that human constitution is already made by interactions with cells of different types (and subtypes), displaying diverse, type-associated, activities. 

Moreover, the investigation into the formation of eukaryotic cells strongly suggests a relationship with *mitochondrial* origin and evolution. Famously, mitochondria are sub-cellular organelles essentially to provide chemical energy (in the form of ATP) to eukaryotic cells. Nevertheless, in recent years, it has been shown that mitochondria are also involved in other, crucial, cellular activities, such as metabolism of both amino acids and nucleotides, fatty-acid assimilation, protein synthesis, forms of programmed cell death (*e.g.,* apoptosis) and the regulation of various homeostatic factors [24]. However, the fascinating detail is that phylogenetic analyses indicate mitochondria having a prokaryotic origin, thus showing that a fundamental eukaryotic cell compartment has an endosymbiotic provenance. The presence of elements, once exogenous, in the constitution of eukaryotic organisms, should make us reflect on the notions of “identity” and “unity” of biological entities. Accordingly, it should not be strange to look at microbiota as a legitimate part of “humans” as organisms (this somehow implies that if we want to adopt the elusive notion of “human nature,” the microbiota would definitely contribute to it). 

The property of being an *organism* (a central notion in biology [25] is generally associated with the capacity of reproducing within a given specific lineage. *Homo sapiens* (whose genome includes also mitochondrial genome) lineage is notoriously transmitted by vertical descent. However, as recently highlighted, the transmission of the human microbiota to the progeny can also occur through other modalities, less specific but still reproducible. In this way there is the possibility of inheriting a microbiota constituted by a coherent base, specified into inter-individual variations, which are preserved throughout the generations inside a parental line [20]. From this perspective, the various forms of physical-chemical interactions between microbiota and its “host” are so deep-rooted and integrated, that considering microbiota as merely external, just because of the genomic differences, seems, at least, questionable. First, the human genome itself displays the *vestiges* (now fully part of what we call “human”) of past infections and interactions with other organisms (such as the famous Human Endogenous Retro Viruses - HERVs) [26]. Second, the magnitude of activities of the microbiota itself can be genuinely seen comparable to those of a traditional organ [23]. This is also why other authors [27] have proposed to classify (with an eye towards possible manipulation/modification for biomedical purposes in the light of health problems) microbial populations according to *functional criteria* (rather than just phylogenetical). This type of move, not free of problems [28], is somehow motivated by the need of finding how (*e.g.,* in which ways and how much) microbial activity contributes to host functionality despite the distinct genomic origins.

If this is the case, further studies might have the potential to revolutionize entire areas of clinical investigations and the rationale of many health-care strategies. Indeed, certain future therapeutic interventions could be directed to the “new physiological” pathways. Drugs could be designed to address interactions within the “missing organ” itself or with the other ones. As some researchers argue, if medicine has historically been developed through specialties based on organs or organ systems (think of cardiology, pneumology or gastroenterology), it would not be strange to think of a future development of a specific branch such as “microbiomology” or “clinical microbiology.” Such a specialty, starting from the consideration of the structure of the microbiota as an actual organ, would study its physiology and pathology, keeping an eye to its relevance both in diagnostics and preventive medicine. [20].

## 4. Beyond the Symbiosis and The Missing Organ: the “Holobiont”

By considering the countless interactions between the host and its microbiota (however differently composed) either as a symbiosis or as a relationship between an organism and one of its organs is still far from a radical conceptual change that some scientists are proposing in addressing these issues.

As already mentioned in the introduction, a completely distinct view that challenges both the theoretical and the experimental side is constituted by the notions of *holobiont* and *hologenome*. According to this perspective [3], we should stop at seeing symbiosis as an interaction (no matter how complex or peculiar) of different organisms (such as the “host” and its commensal associated species) but rather as a complex unity of biological organization (as much as the organism itself) that should be considered as the privileged one. The specificity of this viewpoint is that, contrary to the traditional notion of symbiosis, which is basically an extension of the current understanding concerning these phenomena, the notions of *holobiont* and *hologenome* challenge the contemporary conceptual view. They do so by offering not just a different angle from which one should look at these phenomena but a deconstruction of the categories according to which we consider those “things,” precisely our phenomena on interest (*i.e.,* the system we want to investigate). Moreover, such a change of prospect will inevitably provide different categories and, as a consequence of that, a different experimental possibilities. As François Jacob famously argued, the work of the biologist necessarily starts with the choice of an operative framework, defined as the “experimental system.” According to Jacob, all depends from this initial decision: the type of experiments that can be conducted but also the type of legitimate hypotheses that can be formulated and therefore also the type of answers the scientist can obtain [29]. According to this sense, due to their hybrid nature (in between organisms and communities) [30], holobionts and hologenomes might have the potential to become a “New System.” 

Bordenstein and Theis [3] also provide a list of key points in order to clarify the holobiont perspective and to avoid misconceptions about it.

First, as already mentioned, holobionts and hologenomes can be seen as units of biological organization. This immediately embeds an ecological perspective in the study of molecular interactions. Being a unit of biological organization means to understand that organisms do not exist in isolation. From a functional point of view, their existence, development, evolution are intrinsically shaped by the reciprocal interaction and by the relation with the environment (both organic and not organic). For instance, one can see a virus as simply as a sequence of either DNA or RNA within a protein capsid. But from a different viewpoint, let us call it a *processual view*, the perspective of biological organization around units formed by different organisms and their genomes, that virus is now a part of interconnected system to which it will contribute in terms of physiology, development, function or dysfunction. 

Second, it is important not to mistake holobionts and hologenomes for organs, super-organisms or metagenomes. Indeed, on one hand, organs and super-organisms both share the same genomes, while on the other, the term metagenome simply states that there is something more than the genome but clearly does not take into account the interactive/organismic/ecological perspective, which is at the core of holobiont and hologenome conceptualization (as the “disunity” of the holobiont brings important features as much as its unity [30]).

Third, the netlike nature of hologenome suggests that all the genomes involved should be considered. Moreover, being hologenomes a multiplicity of genomes (nuclei, organelles, bacteria, fungi and viruses), variations and mutations can occur at any level of the network and might have an impact on the entire organizational unit. In addition, since such units could be the target of evolutionary change, this change in perspective might also affect our perception and conceptualization of the evolutionary process itself. This should not be taken in a radical, superficial or simplistic way, meaning that we need to abort the *evolutionary theory*. Rather, both hologenomes and holobionts challenge a particular interpretation of evolutionary change, the so-called “neo Darwinian framework,” which neglects any form of plasticity or porosity within the genome itself. On the contrary, by adopting a view closer to, the so called, “extended synthesis” [31,32], both inheritance and different forms of genetic transmissions such as horizontal gene transfer [2] (which is now documented also between prokaryotes and eukaryotes) as well as the incorporation of environmental elements, should be now fully considered. 

## 5. Holobionts, Faecal Transplantation and Bacteriotherapy.

The holobiont perspective is particularly intriguing (and challenging) when specific therapeutic interventions concerning the microbiota are considered. Nowadays, Faecal Microbiota Transplantation (FMT) is a quite established therapeutic intervention, famously adopted to treat infection with *Clostridium difficile* [33,34] In the wake of this success, it has been suggested that the microbiota could be seen and adopted as a real therapy, a *bacteriotherapy* [33], also in other pathological conditions. Again, from a conceptual point of view, this approach poses this type of intervention as a hybrid, in between “organ transplantation” and “immunotherapy.” 

From the holobiont perspective the possibility to use microbiota as a therapy (and a personalized one, see also section 8) can be seen as a way to re-establish/reinforce either the structure or the efficiency of a network whose functionality and shape are at core of the global health of the system. Thus, several approaches may be adopted to pursue this scope (mimicking traditional strategies). On the one hand, dietary and lifestyle interventions (*e.g.* probiotics, prebiotics, other nutraceutical compounds) can directly modulate the composition and the functionality of microbiota [35,36,37,38]. Other approaches involve a genetic manipulation of microbiota itself [36,39,40]. In this case the idea is to use genetically modified strains of bacteria in order not only to act as ad hoc therapy but also in the preventive phase, as tools to diagnose specific pathologies. By trying to intervene on the network (*i.e.,* intervening on the holobiont), this approach has the potential of being more effective, more coordinated and, at the same time, less invasive and less expensive compared to current methods [39].

However, despite the coherence of the hypothesis, the novelty of the field and the consequent lack of knowledge both pose some difficulties that cannot be forgotten. As a matter of fact, an increasing number of studies do suggest that the microbiota is directly involved in the metabolic syndrome, showing a role in determining key factors such as insulin resistance or blood pressure [33]. Nonetheless, at the moment, only few researches show sufficiently robust data to legitimize a direct causal link (on which researchers and clinicians might operate). Therefore, some researchers have highlighted the need to investigate these relationships through new studies (including RTCs) that require greater attention to the specific bacterial strains involved, to the changes in their metabolites, trying to clarify how the genetic and epigenetic aspects of the microbiota and the host will influence each other (of course the relationship with the immune system is central here) [10,13,33]. 

Moreover, as recently reported and coherently with the holobiont perspective, the microbiota appears to be deeply personalized, showing a high inter-variability among different subjects (also, due to dietary diversities, showing variations among cultures and geographical regions) and important fluctuations during diverse phases of the day [13]. In addition, in case of genetically engineered microbiota, despite their designed precision, the impact of their introduction on the entire network (with possible unwanted effects) still needs to be fully addressed and evaluated.Nevertheless, these difficulties should not discourage further scientific investigations. In the recent years we have understood that the interactions between the elements and the components of that system that is our organism (and on this see also section 6) are more complex than we thought. At the same time, this awareness shows us the need for a new systemic viewpoint (and the limitation of purely reductionist approaches). The fact that many aspects of this perspective are still obscure is precisely a reason to conduct more research into it.

## 6. The Polygenomic Organism

The increasing interest towards microbiota and its interactions (whose modalities are still widely unknown) and the rise of new concepts such as holobionts and hologenomes (to deal with the netlike nature of these phenomena) urge us to critically address the notion of organism itself which was already at the centre of conceptual change in biology [25,41]. However, this is not because of a highly theoretical, speculative exercise (that, nevertheless, will be interesting in itself). Rather, by reconsidering fundamental notions at the core of the theoretical apparatus we adopt to deal with disease and health we might find new ways to approach issues that really matter from a practical point of view.

A first way to challenge the current concept of organism is the assumption of individual *genetic homogeneity*. According to some scholars this idea is decidedly deceptive. In fact, such a view constitutes the basis of a series of misunderstandings concerning the characteristics of those systemic entities, usually called as “organisms [42]. 

The first critical element of genetic homogeneity is understanding that in both natural and artificial processes we see a significant degree of chimerism and mosaicism in many, not to say almost all, multicellular organisms, including humans. The degree of similarity in this context seems to be fuzzier than previously described. Therefore one may argue that the hypothesis according to which every cell of an organism has the same genome of another one could be, at least, an oversimplification of a more intricate picture [42].

A second layer is constituted by *epigenetics*. Epigenetics has shown that, even if genomes of cells within an organism will be somehow homogeneous in their constitution, the way in which genes and other functional elements of a genome are expressed is far from being the same. Rather, such a diversity in expression and regulation provides also an explanation of different cell types [42].

By following these suggestions, it is possible to conceive that entities we classify as living unities might not be those identified by our traditional notion of species and organisms. For instance, the phenomenon of chimerism challenges our ideas about the consistency of organisms themselves. Moreover and maybe more dramatically, the profound interactions and interconnections among living beings normally considered members of the different species or lineages. Of course, the change of perspective is not meant to determine which should be the right way to divide and classify the living world. Rather, it suggests that different approaches might reveal connections and causal relations that may result intrinsically hidden, according to other ways of partitioning the living world.

As is well argued by Dupré, the different elements of a cell can “co-operate” in a rather complex way. In some cases, the combination of different communities of different species of microorganisms gives rise to real diversified and separate lineages (such as mitochondria). In other cases, the cooperation characterizes phenomena of even higher complexity (the case of multicellular organisms) and we face real *systemic units*, composed of different entities, interacting together in a synergistic way. A situation so complex and articulated between cooperative and competitive processes, will also require that different research interests, bearers of different questions, would be and should be prosecuted. It is not bizarre to think that such research directions will produce new ways of delimiting and distinguishing biological individuals and their boundaries, or at least to believe they will deeply question those currently employed [42].

## 7. Microbiota and Health: the Ecological Perspective and New Research Directions

If we take all these perspective seriously, it is hard not to believe that they are going to affect other notions, central to both research and clinical investigation. If the notion of biological individual will be modified (or at least updated), also the concept of health itself may face a radical change. In other words, if we move from the conception of individuals as *punctiform units* to the view that what we call individuals are rather *peculiar intertwined networks*, or sophisticated micro-ecosystems, the idea of health seems to acquire a more ecological nuance [43], also suggesting a complex system of *health levels* in which that individual health cannot be fully detached from “healthy conditions” of other layers into play. 

This is not just in theory. In the last years, several promising, interdisciplinary approaches have been developed to translate an ecological perspective into research and clinical practices. For instance, in the case of cancer, increasing studies suggest how it should be considered as a multifactorial disease [44,45]. This is due to the fact that a large number of endogenous and exogenous factors (such as diet, exposure to certain environments, lifestyles) and their peculiar (most of the time *personalized*) combination, can give rise to the conditions for the development of the disease. 

In this respect, immunotherapy represents a promising action line. However, if the microbiota is actually considered as a functional part of the immune system itself and thus, the immune response is a network-based, environmental feature, the ecological perspective becomes central in analyzing the immune response in the tumour microenvironment [45]. Moreover, if we embrace a network perspective on health (such as the holobiont view) the distinction of exogenous and endogenous becomes less neat and less sharp.

The emerging field of *molecular pathological epidemiology* (MPE) constitutes a promising research agenda in this direction, by creating a hybrid field between pathology and data science [46,47]. This approach promotes a view that combines various potential risk factors (the epidemiological point of view) with molecular pathology [47]. Accordingly, any disease can be phenotyped, based on pathogenic mechanisms. A research based on MPE can determine a better understanding of pathogenic landscape and evaluate how an association is depth and intense for given subtypes. Unlike traditional epidemiological research, this approach may help detecting causal connections [46]. This is particularly relevant, given that microbiota’s activities and functions, by playing potential diverse (protective or risk) factors, can be finally seen in the web of causal connections, thus determining the overall health status of the network. 

Given the current scope of personalized/precision biomedicine in treating the individual, rather than disease, the transformation foreseen here may also change the strategies we adopt in dealing with a various number of pathologies, still not fully curable or treatable.

## 8. Precision Medicine/Personalized Medicine: the Individual and the System

**Figure 2:**
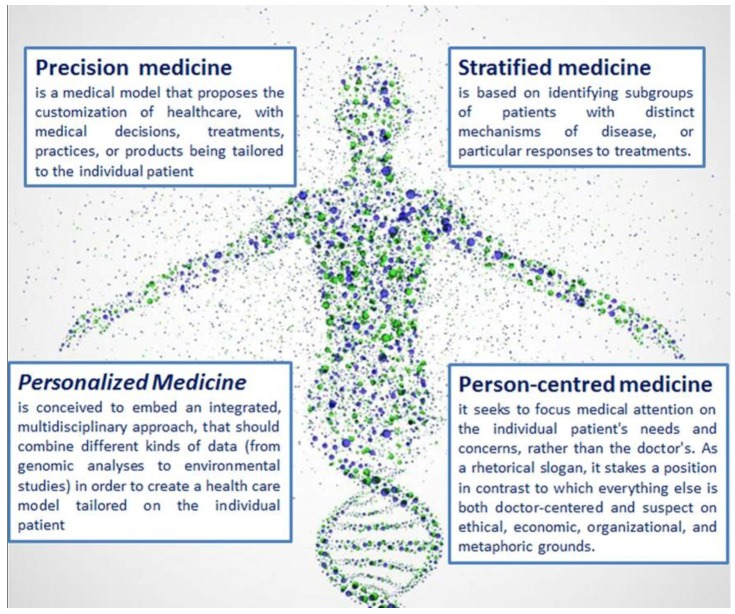
Definition of personalized, stratified, person-centred, precision medicine. We have fine defined these expressions that are often used interchangeably.

In the last years, the health care system has rapidly changed. Both newspapers and specialized press report and hail the new era of personalized, stratified, person-centred, precision medicine (Figure 2). Indeed, these expressions are often used interchangeably [48]. This semantic blurriness [49] mirrors economic, institutional solutions and approaches, aiming at the establishment of a new paradigm for health care systems.

Spanning from the molecular understanding of biological phenomena to the implement of computational resources to study the general behaviour of living systems, *precision medicine* is normally conceived to embed an integrated, multidisciplinary approach, that should combine different kinds of data (from genomic analyses to environmental studies) in order to create a health care model tailored on the individual patient [50]. On the other hand, new approaches and techniques have fostered the division of population into many subgroups, which eventually need different and specific treatments (the so-called stratified medicine), thus paving the way to the decomposition of the universality of human kind, into a myriad of unique variants that will be able to detect individual specificities and therapeutically address them: personalized medicine. 

Despite this terminological struggle, what is at stake here is the individuation of those features that contribute to the creation of medical model that should be more *predictive*, more *preventive* and more *personalised* (also in the sense of paying more attention to those aspects, cultural, ethical and psychological, often neglected by the clinic dimension). Therefore, a Predictive, Preventive and Personalised Medicine (PPPM) [50] will try to focus on diverse layers and aspects that contribute to dissect the notions of individual, health and disease and relative interventions. Thus, according to this perspective, both genetics and epigenetics, life style and related activities (such as diet and sport), socio-economic conditions, cultural belonging and psychological setting (all elements on which the impact of microbiota can be critical and cannot be mistreated) should all receive full consideration in a commonly framed manner [50]. Moreover, other aspects such as translational research, information technology and public dissemination of scientific and clinical results, biolaw and education will take part into the new model. 

At first glance big data analyses granted by computational/omics methods seem to go in the direction of fostering this approach through the capacity to deal with data and information coming from various sources. However the situation might not be so optimistic. The case of precision oncology is somehow emblematic. In a recent issue on Nature, the haemato-oncologist Vinay Prasad has revealed that a large number of cancer patients has not benefited from the so-called precision medicine. Although the genome of a large number of patients (tens of thousands) has been already sequenced, the number of interesting or significative cases remains extremely low. Moreover, this approach does not seem to have shown that it can improve the results in controlled studies. Therefore precision oncology remains, as such, an approach still waiting to be corroborated and established [51]. How is it so?

Here of course we cannot and should not provide a technical answer. However it seems that profiling patients, in the light of precision/personalized medicine approaches, does not provide what has been thought to promise, probably due to the fact that the “complexity” of a patient’s individuality (affecting also its clinical condition) may not and cannot be simply reduced to the collection of its data (or represented by it), no matter how much precise and rigorous such analysis can be. This is not to say that computational strategies employed by precision-oncology are useless. On the contrary, beside the information they provide concerning human genetic variants and stratification, they were also extremely important in revealing that personalization of healthcare (both in terms of precision and consideration of specific patient needs) cannot be done simply by enlarging the amount of data nor the sources of data. The determinants of individual health, which contribute to the specific identity of a single patient, cannot be fully and correctly understood just in terms of omics analysis.

By considering what has been said, it seems that biomedicine is facing a sort of paradox. On the one hand, both scientific understanding and patients’ needs push towards a focus on the individual. On the other hand, research findings suggest that the decomposition of biological phenomena and their (so-called) reductionist analysis are only methodological and require more and more a systemic perspective in order to be adequately addressed and explained [52]. In addition, not only does traditional medical taxonomy (organ based) seem no longer suitable to fully appreciate the novelties of current scientific understanding of disease and health (and thus the potential therapies) [53] but the patient themselves, the biological entities that are the biomedicine focus, cannot longer be seen just as single units or “monads,” bottom-layers of individualization.

In this respect, we propose that the advancement of the studies on the microbiota (both conceptual and empirical) may suggest a way to reconcile diverse perspectives and somehow to “solve” the paradox. Indeed, by looking at individual patients as networks of different but nevertheless fundamental, components it could be possible to start thinking and designing new systemic approaches for therapy that will be tailored but not reductionist. By forcing us to see human beings as ecosystems, these studies will help, somehow surprisingly, to better understand the individual patient and, at the same time, they will fully acknowledge that those individuals are also, by some means, a “multitude.”
